# Influence of Selectively Localised Nanoclay Particles on Non-Isothermal Crystallisation and Degradation Behaviour of PP/LDPE Blend Composites

**DOI:** 10.3390/polym10030245

**Published:** 2018-02-28

**Authors:** Tladi Gideon Mofokeng, Suprakas Sinha Ray, Vincent Ojijo

**Affiliations:** 1 DST-CSIR National Centre for Nanostructured Materials, Council for Scientific and Industrial Research, Pretoria 0001, South Africa; TMofokeng@csir.co.za (T.G.M.); VOjijo@csir.co.za (V.O.); 2 Department of Applied Chemistry, University of Johannesburg, Doornfontein 2028, South Africa

**Keywords:** blend, nanoclay, compatibility, nucleation, activation energy

## Abstract

In immiscible polymer blend nanocomposites, nanoparticles can be localised either in polymer matrices or at the interface, invoking the simple question of how the spatial distribution of the nanoparticles and the resulting morphological changes affect the non-isothermal crystallisation and degradation kinetics. In this study, the non-isothermal crystallisation of polypropylene in polypropylene (PP)-rich compatibilised and non-compatibilised PP/low-density polyethylene (LDPE)/clay composites and their degradation are investigated. The non-isothermal crystallisation analyses show that the localisation of the clay particles in the blend composites has two opposing effects. First, the poorly dispersed clay particles at the PP/LDPE interface in the non-compatibilised blend composite has no significant effect on the crystallisation temperature of PP but allows the free movement of PP chains, resulting in a higher crystallinity of PP than that of PP in the neat blend. Second, the well-dispersed clay particles in the compatibilised blend composites disrupt the free movement of PP chains, resulting in a lower crystallisation temperature and crystallinity than that of PP in the neat blend. The influences of different selective localisations of clay particles on the activation energies of degradation are studied. The presence of maleated compatibilisers, clay, and the distribution of clay in the blend composite play important roles in determining the activation energies of degradation.

## 1. Introduction

For many decades, polymer blends have attracted attention because the process of mixing two or more polymers with different physical properties provides a simple route for tailoring polymer properties for suitable applications. Until recently, blends of polypropylene (PP) and polyethylene (PE) have been the subject of ongoing research for many years due to the remarkable properties of the blended polymers [1,2,3,4,5,6]. PP has fascinating properties such as excellent chemical resistance, processability, and a relatively higher stiffness. However, its inadequate flexibility limits its use for certain applications. Therefore, the melt blending of PP with a relatively flexible polymer such as low-density polyethylene (LDPE) might improve the flexibility of PP. The process of blending PP with LDPE to enhance its flexibility might sacrifice the stiffness of PP, thereby limiting its versatile application to some degree. Thus, compounding the blends of PP and PE with inorganic nanofillers such as nanoclay to form a blend composite is a simple and cost-effective method of enhancing the mechanical and thermal properties.

In polymer composites, the polymer crystallisation process may have a significant influence on its morphological features and thermal and mechanical properties. It is essential to study the non-isothermal crystallisation kinetics of semi-crystalline polymers in polymer composites because industrial production processes are usually carried out under non-isothermal conditions. The knowledge of non-isothermal crystallization kinetics is essential to achieve the proper microstructure and the required properties of a polymeric material [7].

Numerous authors [8,9] have investigated the crystallisation behaviour of melt-mixed PP/clay composites using non-isothermal crystallisation kinetics and different models to determine the activation energy of crystallisation. Generally, the authors [8,9] reported that the method developed by Mo and co-workers is successful for describing the non-isothermal crystallisation kinetics of PP and PP/clay composites. Moreover, the clay particles had a nucleation effect on the crystallisation of PP for PP/clay composites. Nagendra et al. [10] prepared PP/clay composites using unmodified layered double hydroxide (LDH) via two methods of solution mixing. In the first method, the gel form of LDH was directly dispersed in a solution of isotactic PP to prepare highly dispersed nanocomposites, whereas the second method involved dispersing LDH, which was sonicated for 4 days in isotactic PP. The authors [10] reported a better nucleation ability for PP in sonicated LDH containing nanocomposites than when un-sonicated LDH is used. They attributed their results to the high specific surface area of the sonicated LDH clay particles and their better dispersibility in PP in comparison to using un-sonicated LDH.

However, the better dispersion of clay in PP/clay composites does not always guarantee better nucleation of PP. Lai et al. [11] investigated the dispersion of organoclay (C20A) in PP and the physical properties of the nanocomposites produced in the presence of compatibilisers, polyethylene-octene-elastomer-grafted maleic anhydride (POE-*g*-MA) and maleated polypropylene (PP-*g*-MA). Compatibilisers are block copolymers, graft copolymers, reactive polymers [12], and nanoparticles [13] that are incorporated in immiscible blends to strengthen the interfacial interactions and reduce the phase coalescence. In polymer/clay nanocomposites, maleated polymers act as compatibilisers by enhancing intercalation between clay and polymers [14]. The authors [11] reported a higher crystallisation temperature (*T_c_*) for PP when either PP-*g*-MA or POE-*g*-MA was added to PP, suggesting that the compatibilisers serve as nucleating agents. However, *T_c_* of the PP/clay nanocomposite was equal to that of neat PP, whereas the PP/PP-*g*-MA/clay and PP/POE-*g*-MA/clay nanocomposites, despite containing better dispersed clay particles, had lower values of *T_c_*. The authors [11] attributed these lower values of *T_c_* to the shielding, plasticising, and/or miscibility effects of the compatibilisers.

Bandyopadhay and Ray [15] investigated the effects of nanoclay on the non-isothermal crystallisation kinetics of PP in blend composites containing poly[(butylene succinate)-*co*-adipate] as a minor phase. The authors [15] reported that the Avrami and Liu models were successful in describing the non-isothermal crystallisation kinetics of their samples and that the blend composite had a higher activation energy of crystallisation than both neat PP and the blend, suggesting the slow crystallisation kinetics of PP in the presence of nanoclay. Goodarzi and colleagues [16,17] established correlations between the morphology and both the thermal degradation and crystallisation of PP/ethylene vinyl acetate/clay composites.

The distribution and dispersion of nanofillers in immiscible polymer blends are two of the well-known developmental steps of polymer technology. The selective localisation of nanofillers in different polymeric phases of blend composites may influence the crystallisation and thermal degradation kinetics differently, and inadequate work has been carried out on this topic. Given this background, we have previously melt-mixed clay containing PP/LDPE blend composites in the absence and presence of compatibilisers, PP-*g*-MA and maleated polyethylene (PE-*g*-MA), and investigated how the localisation of organically modified nanoclay particles affects the morphological development—and hence the properties—of the obtained composites [18]. Nanoclay was used as nanofiller to produce the blend composites due to its significant property enhancement at low loading, commercial availability, cost effectiveness, and relatively simple processability [19]. The PP-*g*-MA and PE-*g*-MA were selected because they contain polyolefin groups which are miscible with the PP and LDPE phase of the blend, respectively. The polar hydrophilic maleic anhydride groups of PP-*g*-MA and PE-*g*-MA would interact with the polar groups on the clay surface to improve the dispersion/distribution of nanoclay platelets in PP and LDPE. The results showed that the nano/microstructure of the PP/LDPE blend can be controlled by incorporating nanoclay alone, which selectively localised at the interphase of PP and LDPE. Moreover, the addition of mixtures of organoclay and PP-*g*-MA, organoclay and PE-*g*-MA, and organoclay with a mixture of compatibilisers to the PP/LDPE blend in each case manipulated the localisation of clay particles to the PP major matrix phase, LDPE minor phase, and all phases of the PP/LDPE blend, respectively.

Therefore, the relevant question is how do clay localisation and its spatial distribution in different phases of the PP/LDPE blend affect the crystal growth of the PP matrix during non-isothermal crystallisation? From the viewpoint of fabricating ternary nanocomposites with tuneable properties, the main objective of this study is to investigate the influence of the localisation of clay particles in an (80/20) PP/LDPE blend and its resulting morphology on the non-isothermal crystallisation of PP and the kinetics of thermal degradation in the blend composite. It will be shown that there is a correlation between the microstructure of the blend composites and the crystallisability of PP.

## 2. Experiment

### 2.1. Materials

LDPE and PP were commercial grades with undisclosed molecular weights, but low MFIs. PP-*g*-MA and PE-*g*-MA were also commercial grades commercial grades with undisclosed molecular weights, but relatively higher MFIs. LDPE and PP were purchased from Sasol, South Africa. The melt flow indices (MFIs) of the polymers were determined using a CEAST melt flow monitor multi-weight protocol. The LDPE used in this study is commercial-grade (LT033) with an MFI of 2.25 g per 10 min (230 °C/2.16 kg). PP is commercial-grade (HHR102) with an MFI of 3.41 g per 10 min (230 °C/2.16 kg). The organically modified montmorillonite (OMMT) used in this study is Cloisite^®^ 20A, purchased from Southern Clay Products, Inc., Gonzales, TX, USA. This compound is an MMT modified with a dimethyl dihydrogenated tallow quaternary ammonium salt. The PE-*g*-MA compatibiliser with an MFI of 121.9 g per 10 min (100 °C/2.16 kg), and 0.5 wt % MA was purchased from Sigma-Aldrich, Inc., St. Louis, MO, USA. The PP-*g*-MA compatibiliser (commercial-grade VINBOND) with an MFI of 58.31 g per 10 min (190 °C/2.16 kg), and 1 wt % MA was purchased from VinPoly Additives Pvt. Ltd., New Delhi, India.

### 2.2. Sample Preparation

OMMT, PP-*g*-MA, and PE-*g*-MA were dried in a vacuum oven at 80 °C overnight prior to processing. The weight ratios of PP to LDPE in the neat blend and ternary composites were maintained at 80:20 because in a previous study [6], this blend composition provided balanced mechanical properties. The compatibiliser and OMMT ingredients of the samples remained at 5 and 4 wt %, respectively. The designations and compositions of the samples are listed in Table 1. All of the ingredients of the samples were simultaneously extruded using a twin-screw extruder. The heating zones of the extruder were set at temperatures in the range of 120–180 °C. The extruded samples were quenched in tap water, pelletised, and dried in an oven at 80 °C for 24 h. The dried pellets of the samples were transferred to an injection-moulding machine (ENGEL e-mac50, ENGEL, Schwertberg, Austria) with a 500-kN clamping force to produce a variety of moulded test specimens. The heating zones of the injection-moulding machine were set to 36, 220, 230, 235, and 240 °C. The injection speed, specific back pressure, metering, and injection pressure were set to 220 m·s^−1^, 100 bar, 29 mm, and 270 bar, respectively. The injected specimens were cooled at 25 °C and stored in a Ziploc bag.

Generally, it is known that the maximum improvement in the properties of polymer/clay nanocomposites is obtained at around 2 wt %; whereas, contents of about 6 wt % result in large tactoids which ultimately reduce the mechanical properties. The 4 wt % was selected in this study because it is between 2 and 6 wt %. In future, the influence of clay composition on the non-isothermal crystallization and degradation behaviour will be investigated.

### 2.3. Characterisation Techniques

The samples were annealed at 80 °C for 16 h in vacuum before analysis. Differential scanning calorimetry (DSC) measurements of test samples weighing about 4 mg were carried out using a DSC-Q2000 instrument (TA Instruments, New Castle, DE, USA) in the temperature range of −65 to 200 °C in a nitrogen atmosphere. All samples were tested at the same heating and cooling rate of 10 °C·min^−1^ in three successive scans: heating, cooling, and heating. The thermal history of a tested sample was erased by the first heating, whereas *T_c_* was obtained from the cooling scan. The melting enthalpy (*ΔH_m_*) and melting temperature (*T_m_*) were determined from the second heating scan. The percentage crystallinity (%χ) of the samples was calculated from the results obtained during the second heating scans. The non-isothermal crystallisation kinetics of the samples were also ascertained using the DSC-Q2000 instrument (TA Instruments, New Castle, DE, USA) with a constant nitrogen flow of 50 mL·min^−1^. The samples were first heated from −65 to 200 °C at 10 °C·min^−1^ and maintained at 200 °C for 5 min. After that, the samples were cooled to −65 °C at different cooling rates (*ϕ* = 10, 15, 20, and 25 °C·min^−1^) and then heated immediately to 200 °C at a heating rate of 10 °C·min^−1^. The reported data are representative of three independent tests.

The dispersion of the OMMT in the blend composites was investigated by transmission electron microscopy (TEM, JEM-2100, JEOL, Tokyo, Japan). Thin TEM sections with a thickness of about 80 nm were prepared by cryogenically ultramicrotoming the samples with a diamond knife using a LEICA EM FC6 microtome and stained with osmium.

Polarised optical microscopy (POM) was used to study the spherulite growth and morphology of the molten samples. Samples were sandwiched between two glass cover slips and then heated on a Linkam THMS hot stage (Linkam Scientific Instruments, Ltd., Surrey, KT, UK) from room temperature to 200 °C at a rate of 10 °C·min^−1^, held at this temperature for 5 min, and then cooled at the same rate to 140 °C to allow only the PP matrix component to crystallise. The samples were then held isothermally for 30 min; during the time, images were acquired using POM (Carl Zeiss imager Z1M, Carl Zeiss, Oberkochen, Germany). The non-isothermal degradation experiments were performed in air in a TG analyser (model Q500, TA Instruments, New Castle, DE, USA) with samples weighing about 10–15 mg. The samples were heated from room temperature to 750 °C, and heating rates of 5, 10, 20, and 30 °C·min^−1^ were used.

## 3. Results and Discussion

### 3.1. Melting and Cooling Properties

**Figure 1:**
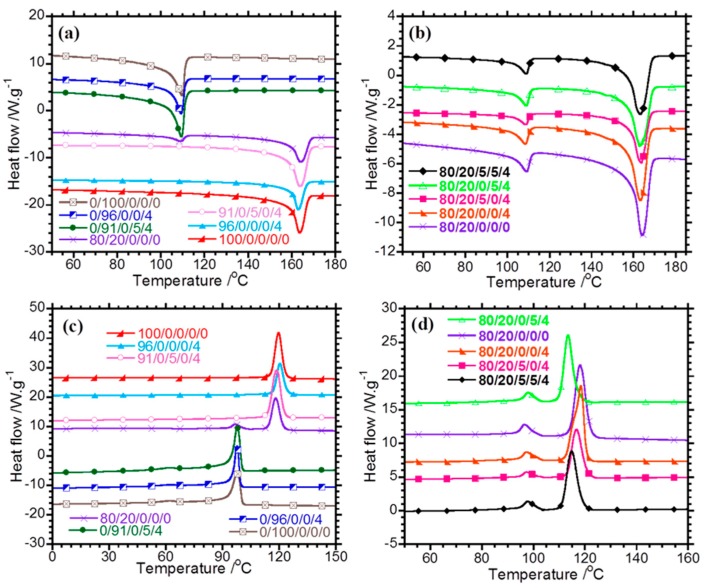
(**a**,**b**) Melting and (**c**,**d**) cooling curves of the neat polymers, blend, and composites.

**Figure 2:**
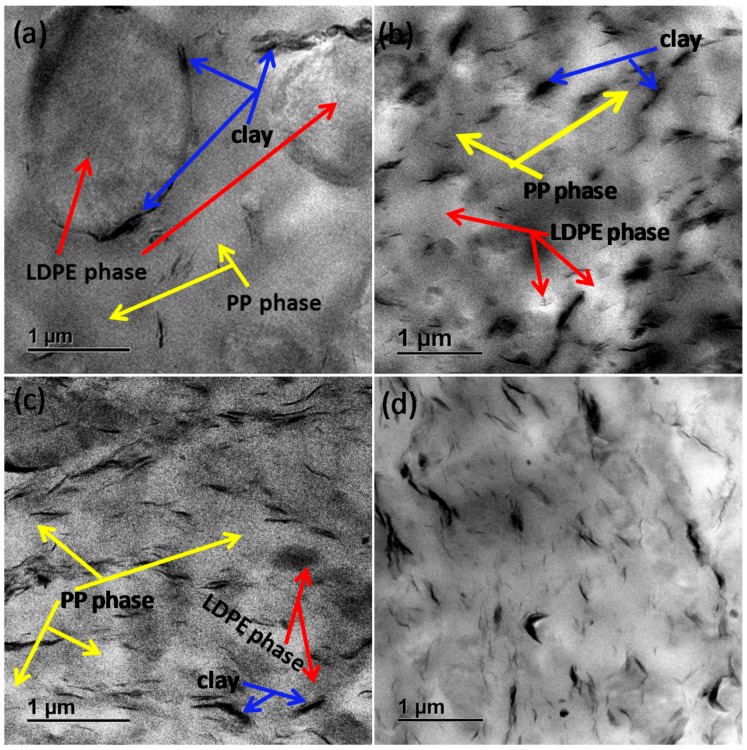
TEM images of PP/LDPE/PP-*g*-MA/PE-*g*-MA/clay: (**a**) 80/20/0/0/4, (**b**) 80/20/5/0/4, (**c**) 80/20/0/5/4, and (**d**) 80/20/5/5/4. The arrows indicate PP phase, LDPE phase, and nanoclay.

The following equations were used to calculate the crystallinity (*χ*) of the polymers in the blends:

(1) $$\%\chi_{pp}=\frac{\Delta {H^{f}}_{m}}{\phi_{PP}\Delta {H^{0}}_{m}\left(PP\right)}\times 100$$

(2) $$\%\chi_{LDPE}=\;\frac{\Delta {H^{i}}_{m}}{\phi_{LDPE}\;\Delta {H^{0}}_{m}\left(LDPE\right)}\;\times 100$$

where *ΔH^f^_m_* is the melting enthalpy of PP, *ΔH^i^_m_* is the melting enthalpy of LDPE, $\%\chi_{pp}$ is the percentage crystallinity of PP, $\%\chi_{LDPE}$ is the percentage crystallinity of LDPE, *ΔH*^0^*_m_*(*LDPE*) is the equilibrium melting enthalpy of LDPE = 288 J·g^−1^ [20], *ΔH*^0^*_m_*(*PP*) is the equilibrium melting enthalpy of PP = 207 J·g^−1^ [21], *ϕ_PP_* is the weight fraction of PP, and *ϕ_LDPE_* is the weight fraction of LDPE. In Figure 1a, neat PP has melting temperature (*T^f^_m_*) of 163 °C, whereas the melting temperature of LDPE (*T^i^_m_*) is 109 °C. The 80/20/0/0/0 blend has two melting peaks associated with the melting of the individual polymers. The melting temperatures of PP and LDPE in the binary (Figure 1a) and ternary (Figure 1b) composites are the same as those of neat PP and LDPE, suggesting that the thickness of the polymer crystals is not affected by the incorporation of clay and its spatial distribution in different phases of the polymeric part of the composites. The clay particles in the composites are confined to the amorphous phase and do not affect the development of crystals in the polymer matrix. The clay particles can also decrease or increase the crystallisation rate by acting as nucleating agents. In Figure 1c and Table 2, PP has a crystallisation temperature (*T^f^_c_*) of 119 °C, whereas neat LDPE has two crystallisation peaks at 97 and 61 °C. The larger crystallisation peak of LDPE at 97 °C is attributed to primary crystallisation into thick lamellae (*T^i^_c_*), whereas the smaller peak at 61 °C is attributed to secondary crystallisation into thin lamellae. The incorporation of clay into PP and LDPE has no effect on *T^f^_c_* and *T^i^_c_*, and this can be attributed to the poor interactions between the modifier of clay and the polymers. PP and LDPE are non-polar in nature and form poor or no interactions with the polar silicate layers of clay in PP/clay and LDPE/clay composites. *T^f^_c_* of PP in the 80/20/0/0/0 blend is the same as that of neat PP, suggesting that an LDPE content lower than 20 wt % is not sufficient to induce a change in the peak position of *T^f^_c_*. In the previous studies [6,22], it was reported that the incorporation of LDPE into PP reduces the crystallisation temperature of PP. The 80/20/0/5/4 nanocomposite has the lowest *T^f^_c_* of the ternary nanocomposites, and this might be due to its higher total mass fraction of LDPE, as observed in Table S1 taking into consideration the presence of PE in PE-*g*-MA. From Figure 1d and Table 2, it is observed that *T^i^_c_* of LDPE in the ternary composites remains the same, whereas the *T^f^_c_* of PP in the ternary composites changes. In fact, *T^f^_c_* of PP for the 80/20/0/0/4 nanocomposite is equal to that in the neat blend, whereas the values of *T^f^_c_* of the PP-*g*-MA- or PE-*g*-MA-containing blend composites are lower than that of the neat 80/20/0/0/0 blend. These results suggest that there is a link between the microstructure of the blend composites and the crystallisability of PP therein. The crystallisabilty of PP in the blend composites is influenced by intervention of nanoclay and dispersion of the LDPE phase. Briefly, the TEM images in Figure 2 of the PP-*g*-MA and PE-*g*-MA-containing blend composites show well-distributed and dispersed clay particles in the polymeric phases, and more details can be found in our previous study [18]. In addition, the LDPE phase is better dispersed in the PP-*g*-MA and PE-*g*-MA-containing blend composites due to combined interventions of clay and maleated compatibilisers. Therefore, the clay particles in the PP-*g*-MA and PE-*g*-MA containing blend composites serve as anti-nucleating agents, which provide enhanced hindrance of the chain mobility of PP; hence, *T^f^_c_* is lowered. Moreover, the LDPE phase in the PP-*g*-MA and PE-*g*-MA containing blend composites also contribute to disrupting the chain mobility of PP due to their high surface area to volume ratio. However, incorporating clay alone into the PP/LDPE blend allowed localisation of clay tactoids at the interphase [18], and the LDPE phase of the 80/20/0/0/4 composite has a relatively lower surface area to volume ratio. Therefore, the poor dispersion of clay tactoids and LDPE phase in the 80/20/0/0/4 composite allow chain mobility of PP; hence *T^f^_c_* is equal to that in the neat blend.

In the binary composites, clay has a greater effect on $\%\chi_{pp}$, whereas $\%\chi_{LDPE}$ is unchanged when clay is introduced. $\%\chi_{pp}$ decreases from 52.8 for PP to 48 for the 96/0/0/0/4 nanocomposite, suggesting that the extent of crystallisation of PP is reduced by the presence of clay. The clay particles in the binary composite form a barrier that retards the development and growth of PP crystals. Regarding the ternary composites containing the maleated compatibilisers in comparison with the neat blend, $\%\chi_{pp}$ and $\%\chi_{LDPE}$ of the ternary composites are lower than those of the neat blend. The simultaneous incorporation of maleated compatibilisers and clay in the ternary composites has promoted better interaction between them and PP/LDPE chains. This is evidenced in Figure 2 by the better distribution and dispersion of clay in the 80/20/5/0/4, 80/20/0/5/4, and 80/20/5/5/4 nanocomposites. As a consequence, this disrupts the chain-folding process and crystallisability of PP and LDPE; hence, $\%\chi_{pp}$ and $\%\chi_{LDPE}$ are lower for the ternary composites containing maleated compatibilisers. The 80/20/0/0/4 nanocomposite has a higher $\%\chi_{pp}$ than the neat blend and other ternary nanocomposites, suggesting increased crystallisability. This is attributed to the observed localisation of clay tactoids at the PP/LDPE interface in Figure 2a promoted by the poor interaction of the clay with either PP or LDPE [18]. Therefore, the clay tactoids at the interface for the 80/20/0/0/4 nanocomposite, which do not interact well with the blended polymers, make it easy for the PP matrix to undergo the chain-folding process. It can be assumed that the localisation of clay at the PP/LDPE interface allows for the easier crystallisation of PP.

### 3.2. Non-Isothermal Crystallisation Kinetics

The Ozawa and combined Avrami–Ozawa models were used to analyse the non-isothermal crystallisation kinetics of neat PP, the blend, and the blend composites. Ozawa [23] and Liu [24] proposed the models expressed in Equations (3) and (4), respectively:

(3) $$\ln[-\ln(1-X_{T})]=\ln X(T)\;-m\ln\phi$$

where *X_T_*, *K*(*T*), and *m* represent the relative degree of crystallinity as a function of the temperature, the Ozawa crystallisation rate constant, and the Ozawa exponent depending on the dimension of crystal growth, respectively, and

(4) lnϕ=lnF(T) −αlnt

where *F*(*T*) refers to the cooling rate required to reach a defined degree of crystallinity, and *a* is the ratio of the Avrami exponent to the Ozawa exponent.

**Figure 3:**
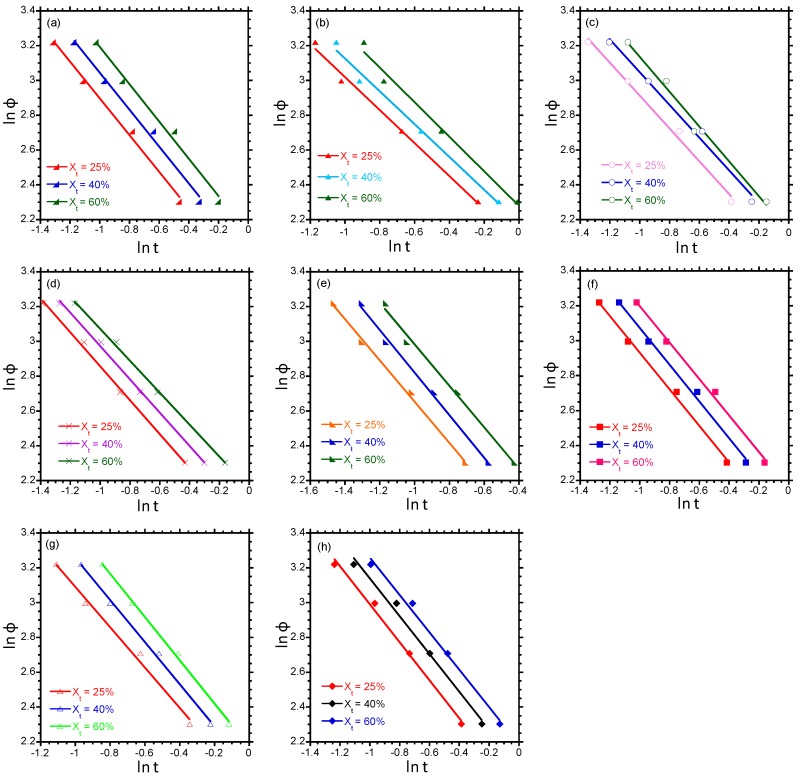
Plots of ln*ϕ* versus ln*t* for PP/LDPE/PP-*g*-MA/PE-*g*-MA/clay: (**a**) 100/0/0/0/0, (**b**) 96/0/0/0/4, (**c**) 91/0/5/0/4, (**d**) 80/20/0/0/0, (**e**) 80/20/0/0/4, (**f**) 80/20/5/0/4, (**g**) 80/20/0/5/4, and (**h**) 80/20/5/5/4.

Plots of ln[−ln(1−*X_T_*)] versus ln*ϕ* and ln*ϕ* versus ln*t* should be straight lines if these models are valid. According to the Ozawa plot (refer to Figure S1), it is generally observed that the Ozawa model fails to describe the non-isothermal crystallisation kinetics of the blend composites. Figure 3 shows that the model proposed by Liu is valid for neat PP, the blend, and the binary and blend composites. The values of *F*(*T*) and *α* were determined from the *y* intercept and slope of the straight lines obtained in Figure 3 and are listed in Table 3. From Table 3, it is observed that there is a systematic increase in *F*(*T*) as the relative degree of crystallinity increases for all samples. For a certain relative degree of crystallinity, the values of *F*(*T*) of the 96/0/0/0/4 and 91/0/5/0/4 nanocomposites are higher than those of neat PP. This indicates that the 96/0/0/0/4 and 91/0/5/0/4 nanocomposites can achieve the same degree of crystallinity as neat PP more slowly, implying that the clay incorporation into PP slows the crystallisation kinetics. With regards to the blend and blend composites, it can be seen that the 80/20/0/0/4 nanocomposite has lower values of *F*(*T*) than the neat blend, indicating that PP in the 80/20/0/0/4 nanocomposite can attain the same degree of crystallinity faster than PP in the neat blend. This suggests faster crystallisation kinetics for PP in the 80/20/0/0/4 nanocomposite and is in agreement with the values of $\%\chi_{pp}$ reported earlier in Table 2, which show a higher $\%\chi_{pp}$ for the 80/20/0/0/4 composite than the blend. Generally, the values of *F*(*T*) of the 80/20/5/0/4, 80/20/0/5/4, and 80/20/5/5/4 nanocomposites are slightly higher than that of the neat blend. This could imply that the presence of PP-*g*-MA and PE-*g*-MA, which improves clay dispersion in the ternary composites, slows the crystallisation kinetics of PP.

### 3.3. Calculation of the Activation Energy for Non-Isothermal Crystal Growth

The activation energy (*∆E*) for non-isothermal crystal growth was evaluated from the Kissinger method [25] defined by the following equation:

(5) $$\frac{d[\ln\left(\phi /{T_{c}}^{2}\right)}{d\left(1/T_{c}\right)}=\;-\;\frac{\Delta E}{R}$$

where *R* is the universal gas constant equal to 8.314 J·mol^−l^·K^−1^. A plot of $-\ln\left(\phi /{T_{c}}^{2}\right)$ versus 1/*T_c_* should give a straight line with a slope of $\frac{\Delta E}{R}$; thus, *∆E* is calculated accordingly. From Figure S2, it is observed that the plots of $-\ln\left(\phi /{T_{c}}^{2}\right)$ versus 1/*T_c_* are indeed straight lines, and their *R*^2^ values in Table 4 are greater than 0.98, implying good correlation. From the results in Table 4, neat PP has a higher *ΔE* than both the 96/0/0/0/4 and 91/0/5/0/4 nanocomposites. The lower Δ*E* for the 96/0/0/0/4 and 91/0/5/0/4 nanocomposites indicates that the presence of clay increases the nucleation efficiency. However, the 91/0/5/0/4 nanocomposite has a slightly higher *ΔE* than the 96/0/0/0/4 nanocomposite. A number of studies [26,27] have reported a higher viscosity when a combination of PP-*g*-MA and clay is added to polymers that than when clay alone is added. Therefore, the higher *ΔE* for the 91/0/5/0/4 nanocomposite could be attributed to its higher viscosity promoted by the better confinement of the mobility of the PP chains due to the presence of the clay particles [28].

With regards to the blend and blend composites, the PP-*g*-MA and PE-*g*-MA compatibilised blend composites have a higher *ΔE* than the neat blend. This is attributed to the confinement of the polymer chain mobility due to the presence of clay, which makes it fairly difficult for crystals to form. In general, the PE-*g*-MA- and PP-*g*-MA-containing blend composites have a higher *ΔE* than the 80/20/0/0/4 nanocomposite. This can be linked to the better dispersion and distribution of clay particles in these nanocomposites, which act as physical barriers to retard the growth of spherulites [29]. As a result, the PP-*g*-MA- and PE-*g*-MA-containing blend composites crystallise slower than the 80/20/0/0/4 nanocomposite. The results for *ΔE* are in agreement with the POM images, which will later show the lower spherulite growth rates for the PP-*g*-MA- and PE-*g*-MA-containing blend composites.

### 3.4. Polarised Optical Microscopy

**Figure 4:**
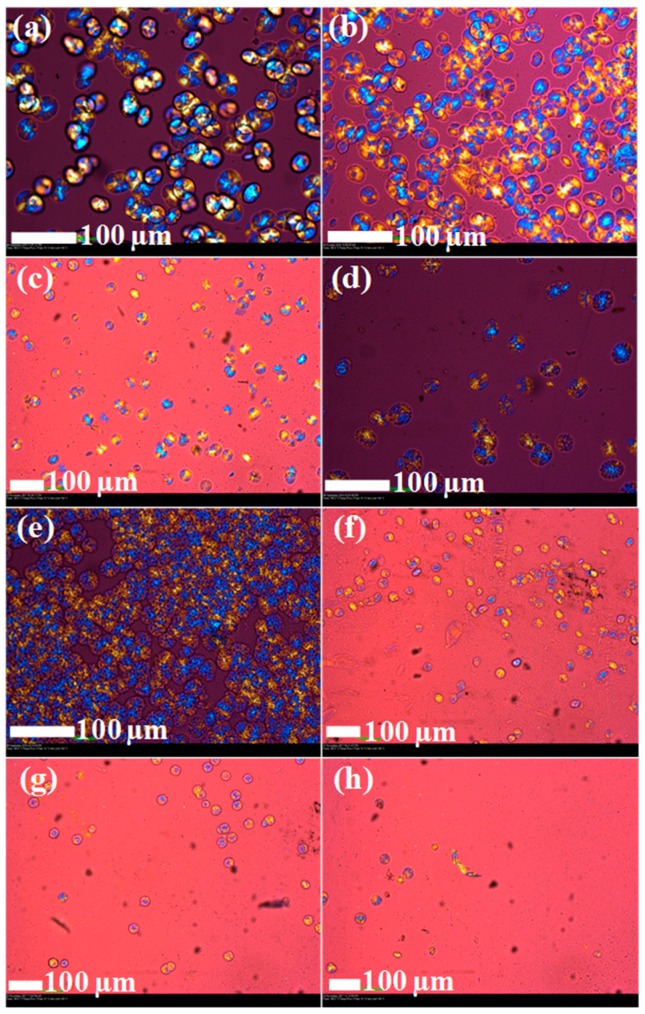
POM images of PP/LDPE/PP-*g*-MA/PE-*g*-MA/clay: (**a**) 100/0/0/0/0, (**b**) 96/0/0/0/4, (**c**) 91/0/5/0/4, (**d**) 80/20/0/0/0, (**e**) 80/20/0/0/4, (**f**) 80/20/5/0/4, (**g**) 80/20/0/5/4, and (**h**) 80/20/5/5/4. The samples were melted at 200 °C followed by isothermal crystallisation at 140 °C for 30 min. The images were taken after 10 min during isothermal crystallisation.

The spherulitic growth rate (*G*) of PP in the samples and its crystalline morphology for the isothermal crystallisation process is presented in Table 5 and Figure 4. From Table 5, neat PP has the highest value of *G*. On the other hand, Figure 4a shows that neat PP has larger spherulites, suggesting that the growth rate of spherulites in neat PP is fast. There is a marked reduction in *G* for PP when clay alone is introduced to PP to produce the 96/0/0/0/4 nanocomposite. This reduction in *G* in comparison with that of neat PP is attributed to intervention by clay on the growth rate of the PP spherulites. In Figure 4b, the number of spherulites increases, whereas their size is reduced when clay is incorporated, suggesting nucleation by clay particles. During injection moulding, the faster nucleation means that the mould can be removed to eject the product at higher temperatures, thus spending less energy on cooling, consequently reducing the cycle time.

In the 96/0/0/0/4 nanocomposite, the presence of clay particles hindered the chain mobility and folding process, resulting in the retarded growth of PP spherulites; hence, a lower *G* is realised. *G* of PP is reduced further with the incorporation of both PP-*g*-MA and clay to produce the 91/0/5/0/4 nanocomposite. The lower *G* for the 91/0/5/0/4 nanocomposite compared with that for the 96/0/0/0/4 nanocomposite is attributed to intervention by PP-*g*-MA on the dispersion of clay. The TEM images in Figure S3 show a relatively better clay dispersion in the 91/0/5/0/4 nanocomposite than that in the 96/0/0/0/4 nanocomposite, which is attributed to PP-*g*-MA, which forms hydrogen bonding interactions between its MA groups and the oxygen groups of the silicate or polar nanoclay surface [14,30,31,32]. Therefore, the relatively better dispersed clay particles in the 91/0/5/0/4 nanocomposite more greatly hindered the chain folding of PP for the growth of spherulites; hence, the lower *G* for the 91/0/5/0/4 nanocomposite in comparison with that for the 96/0/0/0/4 nanocomposite is realised.

A lower *G* and fewer spherulites of PP are observed for the 80/20/0/0/0 blend in Figure 4 compared to neat PP and are attributed to the presence of molten LDPE, which acts as a diluent, thus disrupting the crystallisation of PP when the blend is crystallised at 140 °C. From Table 5, the incorporation of clay into the blend lowers *G* remarkably. The lower *G* of the 80/20/0/0/4 nanocomposite is attributed to the presence of clay at the PP/LDPE interface, which forms a physical barrier that hinders the growth of PP spherulites. The 80/20/5/0/4, 80/20/0/5/4, and 80/20/5/5/4 nanocomposites have lower values of *G* than both the 80/20/0/0/4 nanocomposite and neat blend. The presence of either PP-*g*-MA or PE-*g*-MA in the 80/20/5/0/4, 80/20/0/5/4, and 80/20/5/5/4 nanocomposites resulted in stronger interactions between the polymers and the modifier of clay, promoting better dispersion of clay in the process. As a result, the well-dispersed clay particles in PP-*g*-MA- and PE-*g*-MA-containing blend composites provided better disruption of the chain folding of PP during isothermal crystallisation, resulting in a lower *G*. Therefore, it is deduced that irrespective of the phase at which nanoclay is localised in the blend composites, the crystallisation rate of PP in the blend composites is lower than that of PP in the blend. In addition, improvements in the dispersion of clay in the blend composites using either maleated PP or maleated PE retards the crystallisation of PP; hence, a lower extent of PP crystallisation is realised.

### 3.5. Thermal Degradation Kinetics

**Figure 5:**
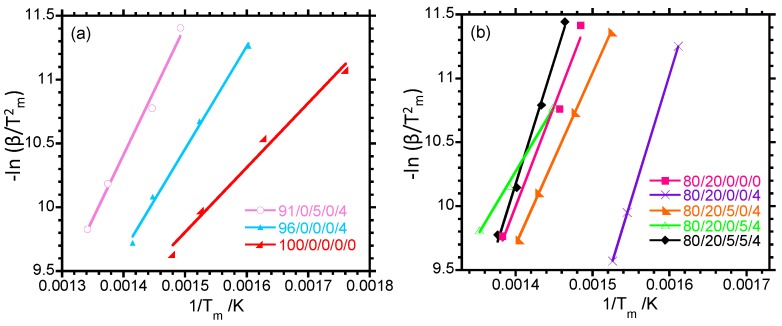
Determination of the activation energy, *E_a_*, describing the thermal degradation process of (**a**) PP and the binary composites and (**b**) the neat blend and ternary composites.

The thermal degradation kinetics of the samples were evaluated to obtain information regarding the degradation behaviour in relation to the localisation of clay in different phases. The activation energy of degradation (*E_a_*) is defined as the minimum energy that is required to initiate a thermal degradation process, and it is related to the temperature dependence of the rate of degradation. The Kissinger method is one of the most common methods that is used to calculate *E_a_* which is calculated as

(6) $$\frac{d\left(\ln\;\beta /T_{m}^{2}\right)}{d\left(1/T_{m}\right)}=\;-\frac{E_{a}}{R}$$

where $T_{m}$ is the temperature of the maximum rate of weight loss obtained from derivative of the thermogravimetric curve. A plot of $-\ln\;\beta /T_{m}^{2}$ versus 1/*T_m_* is a straight line, and *E_a_* can be easily calculated from the slope. The linear curves shown in Figure 5 have *R*^2^ values of 0.98 and 0.99, demonstrating good correlation between the experimental data points and the linear curve. From Table 6, it is observed that *E_a_* = 42.1 kJ·mol^−1^ for neat PP. *E_a_* of PP increases with the addition of clay, which signifies an improvement in the thermal stability of PP. The 91/0/5/0/4 nanocomposite has a higher *E_a_* than the 96/0/0/0/4 nanocomposite, and this can be linked with the better morphology of the 91/0/5/0/4 nanocomposite observed in Figure S3. The well-dispersed nanoclay particles in the 91/0/5/0/4 nanocomposite promoted by PP-*g*-MA also delayed degradation volatiles diffusion out of the polymer during thermal degradation. With regard to the neat blend and ternary nanocomposites, the incorporation of clay into the blend increases *E_a_*, whereas the 80/20/5/0/4 and 80/20/0/5/4 nanocomposites have lower values of *E_a_* compared to the neat blend. In fact, the 80/20/5/5/4 nanocomposite also has a higher *E_a_* than the 80/20/0/0/0 blend. The higher *E_a_* of the 80/20/0/0/4 nanocomposite is attributed to the presence of nanoclay, which prevents the release of the volatiles generated during degradation, which is in line with the increase in thermal stability.

The 80/20/0/0/0, 80/20/5/5/0, 0/0/100/0/0, and 0/0/0/100/0 samples were rheomixed to probe the influence of PP-*g*-MA and PE-*g*-MA on *E_a_* of the 80/20/0/0/0 blend, and the results are reported in the Supporting Information (Figure S4 and Table S2). From the results in Table S2, the 80/20/0/0/0 blend has the highest *E_a_*, whereas PP-*g*-MA and PE-*g*-MA have lower values of *E_a_*. The lower values of *E_a_* for the maleated polymers are attributed to the presence of diacid side groups on those polymers as a result of hydrolysis reactions with the anhydride grafted to PP and PE, which can represent weak sites for the beginning of the decomposition process [33]. It is noted that PP-*g*-MA has a lower *E_a_* compared to PE-*g*-MA, and this is attributed to the higher maleic anhydride grafting in PP-*g*-MA (1 wt % MA compared to 0.5 wt % MA for PE-*g*-MA), suggesting the presence of more diacid side groups. The 80/20/5/5/0 blend (Table S2) has a lower *E_a_* than the 80/20/0/0/0 blend, and this is attributed to the intervention of both PP-*g*-MA and PE-*g*-MA in the lowering of *E_a_* of the blend. In Table 6, the lower values of *E_a_* of the extruded 80/20/5/0/4 and 80/20/0/5/4 nanocomposites in comparison to that of the extruded 80/20/0/0/0 blend are attributed to the intervention by PP-*g*-MA and PE-*g*-MA. This suggests that for these nanocomposites, the effect of compatibilisers in reducing *E_a_* outweighs the effect of the presence of clay in increasing *E_a_*. It is interesting to note that the 80/20/5/0/4 and 80/20/0/5/4 nanocomposites—despite containing well-dispersed and distributed clay particles—have lower values of *E_a_* than the 80/20/0/0/4 nanocomposite, which contains clay tactoids at the interface. This suggests that the effects of the better clay dispersion and distribution on *E_a_* for the 80/20/5/0/4 and 80/20/0/5/4 nanocomposites are also overcome by the intervention of PP-*g*-MA and PE-*g*-MA on reducing *E_a_*. More interestingly, the 80/20/5/5/4 nanocomposite has a higher *E_a_* than the 80/20/0/0/0, 80/20/5/0/4, and 80/20/0/5/4 nanocomposites. The higher *E_a_* of the 80/20/5/5/4 nanocomposite is attributed to the complementary effects of the better compatibility between PP and LDPE promoted by incorporating PP-*g*-MA and PE-*g*-MA and the better distribution of nanoclay in all phases of the 80/20/5/5/4 composite. However, the incorporation of the maleated polymers into the blend reduced *E_a_*, but this effect in the 80/20/5/5/4 nanocomposite is minimal and overcome by the complementary effects of a better compatibility and better distribution of nanoclay.

## 4. Conclusions

The present work investigated the effects of clay localisation and its distribution in an immiscible blend of PP/LDPE on the non-isothermal crystallisation and degradation kinetics. The results showed that there is a correlation between the localisation of clay particles in different phases of the PP/LDPE blend and the non-isothermal crystallisation and degradation kinetics. The incorporation of OMMT into PP and LDPE had no significant effect on their values of *T_c_*, but *T_c_* and the spherulitic growth rate of PP in the PP-*g*-MA- and/or PE-*g*-MA-containing blend composites were significantly lower than that of PP in the neat blend and 80/20/0/0/4 nanocomposite. The same can be said about the neat PP and its compatibilised binary composite in relation to *T_c_* and the spherulitic growth rate. The non-isothermal crystallisation behaviour of the samples was analysed using kinetic models proposed by Ozawa and Liu; the Ozawa model was inappropriate for describing the non-isothermal crystallisation behaviour of blend composites satisfactorily. The non-isothermal crystallisation of the 80/20/0/0/4 nanocomposite showed that the incorporation of clay accelerates the mechanism of PP nucleation, whereas the clay particles in the PP-*g*-MA- and PE-*g*-MA-containing blend composites decelerate it. The much higher activation energies for the non-isothermal crystallisation of the blend composites containing compatibilisers also support slower crystallisation kinetics. The thermal degradation kinetics of the blend composites, which were studied by the Kissinger method, showed that the presence of maleated compatibilisers, clay, and the distribution of clay in the blend composite play a vital role in the resulting value of the activation energy of degradation.

## Figures and Tables

**Table 1:** Compositions of the extruded samples.

PP/LDPE/PP-*g*-MA/PE-*g*-MA/Clay	PP/LDPE Ratio (wt %/wt %)	PP-*g*-MA (wt %)	PE-*g*-MA (wt %)	OMMT (wt %)
100/0/0/0/0	100/0	0	0	0
96/0/0/0/4	96/0	0	0	4
80/20/0/0/0	80/20	0	0	0
80/20/0/0/4	80/20	0	0	4
80/20/5/0/4	80/20	5	0	4
80/20/0/5/4	80/20	0	5	4
80/20/5/5/4	80/20	5	5	4
0/96/0/0/4	0/96	0	0	4
0/100/0/0/0	0/100	0	0	0

**Table 2:** DSC data of pristine PP, LDPE, PP/LDPE blend, and the binary and ternary composites.

Sample	*T^i^_m_* (°C)	*T^f^_m_* (°C)	*T^i^_c_* (°C)	*T^f^_c_* (°C)	*ΔH^i^_m_* (J·g^−1^)	*ΔH^f^_m_* (J·g^−1^)	$\%\chi_{PP}$	$\%\chi_{LDPE}$
100/0/0/0/0	−	163.9 ± 0.3	−	119.1 ± 0.5	−	109.2 ± 1.1	52.8 ± 0.5	−
96/0/0/0/4	−	163.2 ± 0.2	−	120.3 ± 0.1	−	95.4 ± 3.3	48.0 ± 1.7	−
91/0/5/0/4	−	163.9 ± 0.1	−	118.4 ± 0.3	−	96.3 ± 2.0	48.5 ± 1.0	−
0/100/0/0/0	109.6 ± 0.1	−	97.6 ± 0.2	−	130.1 ± 0.7	−	−	45.2 ± 0.2
0/96/0/0/4	109.6 ± 0.2	−	97.5 ± 0.2	−	125.5 ± 5.3	−	−	45.4 ± 1.9
0/91/0/5/4	109.5 ± 0.2	−	97.8 ± 0.2	−	119.8 ± 1.6	−	−	43.3 ± 0.5
80/20/0/0/0	108.7 ± 0.3	163.4 ± 0.6	96.9 ± 0.5	118.4 ± 0.3	17.1 ± 0.9	75.7 ± 2.5	45.7 ± 1.5	29.7 ± 1.6
80/20/0/0/4	108.5 ± 0.1	163.5 ± 0.3	97.4 ± 0.1	118.5 ± 0.2	17.0 ± 0.7	79.1 ± 2.0	49.8 ± 1.3	30.7 ± 1.3
80/20/5/0/4	108.8 ± 0.1	163.5 ± 0.3	97.3 ± 0.1	116.9 ± 0.4	13.2 ± 0.6	70.1 ± 3.5	43.5 ± 2.1	25.2 ± 1.1
80/20/0/5/4	108.8 ± 0.1	162.9 ± 0.2	98.2 ± 0.2	113.6 ± 0.1	16.6 ± 0.7	65.0 ± 1.1	43.1 ± 0.7	24.9 ± 0.1
80/20/5/5/4	108.8 ± 0.1	162.9 ± 0.3	97.6 ± 0.3	114.6 ± 0.2	14.7 ± 1.1	64.5 ± 1.5	42.2 ± 1.0	23.0 ± 1.7

**Table 3:** Kinetic parameters based on the Liu model.

PP/LDPE/PP-*g*-MA/PE-*g*-MA/Clay	Kinetic Parameter	Degree of Crystallinity		
		25%	40%	60%
100/0/0/0/0	*F(T)*	6.298	7.263	8.341
	*α*	1.057	1.062	1.072
96/0/0/0/4	*F(T)*	7.936	8.872	9.761
	*α*	0.948	0.943	0.994
91/0/5/0/4	*F(T)*	7.17	8.213	8.438
	*α*	0.944	0.942	1.003
80/20/0/0/0	*F(T)*	6.563	7.514	8.611
	*α*	0.975	0.956	0.919
80/20/0/0/4	*F(T)*	4.342	4.945	5.995
	*α*	1.186	1.223	1.192
80/20/5/0/4	*F(T)*	6.58	7.54	8.612
	*α*	1.048	1.054	1.046
80/20/0/5/4	*F(T)*	6.905	7.754	8.778
	*α*	1.158	1.209	1.242
80/20/5/5/4	*F(T)*	6.682	7.8	8.894
	*α*	1.091	1.084	1.074

**Table 4:** Activation energies for the overall non-isothermal crystallisation of PP, the blend, and the binary and ternary composites.

PP/LDPE/PP-*g*-MA/PE-*g*-MA/Clay	Kissinger Method	*R*^2^
	*∆E*/kJ·mol^−1^	
100/0/0/0/0	22.5	0.999
96/0/0/0/4	16.7	0.998
91/0/5/0/4	18.8	0.993
80/20/0/0/0	17.2	0.991
80/20/0/0/4	15.3	0.988
80/20/5/0/4	23.5	0.996
80/20/0/5/4	27.0	0.991
80/20/5/5/4	24.7	0.989

**Table 5:** Crystal growth rate of PP of samples isothermally crystallised at 140 °C for 30 min.

PP/LDPE/PP-*g*-MA/PE-*g*-MA/Clay	Spherulite Growth Rate (G), (µm/min)	*T_o_*/min	*R*^2^
100/0/0/0/0	3.23	2	0.998
96/0/0/0/4	2.74	2	0.995
91/0/5/0/4	2.14	2	0.992
80/20/0/0/0	2.93	2	0.994
80/20/0/0/4	2.65	3	0.997
80/20/5/0/4	2.34	7	0.997
80/20/0/5/4	2.41	4	0.997
80/20/5/5/4	2.39	3	0.997

**Table 6:** Activation energies based on the Kissinger method.

PP/LDPE/PP-*g*-MA/PE-*g*-MA/Clay	Activation Energy (*E_a_*)/kJ·mol^−1^
100/0/0/0/0	42.1
96/0/0/0/4	66.7
91/0/5/0/4	83.6
0/100/0/0	139.4
0/96/0/0/4	68.4
80/20/0/0/0	129.9
80/20/0/0/4	163.6
80/20/5/0/4	111.7
80/20/0/5/4	84.2
80/20/5/5/4	160.6

## Supplementary Materials

Supplementary materials can be found at http://www.mdpi.com/2073-4360/10/3/245/s1. Figure S1: ln[−ln(1 − *X_T_*)] versus ln*ϕ* plots for: (a) neat blend and (b–e) blend composites. Figure S2: Determination of the activation energy, *ΔE* describing the nonisothermal crystallization process of PP in neat PP, blend, PP containing binary and ternary composites. Figure S3: TEM micrographs of binary PP/LDPE/PP-*g*-MA/PE-*g*-MA/clay composites. Figure S4: Determination of the activation energy, *E_a_* describing the thermal degradation process of the neat blend, PP-*g*-MA, PE-*g*-MA, PP-*g*-MA and PE-*g*-MA containing blend. Table S1: Amounts of polymers, compatibilizers, and clay present in the samples. Table S2: Activation energy for the overall non-isothermal crystallization of PP-*g*-MA, PE-*g*-MA, PP/LDPE, and PP/LDPE/PP-*g*-MA/PE-*g*-MA blend.
