# Supplementary material for: Influence of Selectively Localised Nanoclay Particles on Non-Isothermal Crystallisation and Degradation Behaviour of PP/LDPE Blend Composites
Source: Polymers (Basel). 2018 Feb 28;10(3):245. doi: 10.3390/polym10030245 (PMC6414920; doi:10.3390/polym10030245)
Supplement: Supplementary file 1 [file polymers-10-00245-s001.pdf]

Influence of Selectively Localised Nanoclay Particles on Non-Isothermal Crystallisation and Degradation Behaviour of PP/LDPE Blend Composites

Tladi Gideon Mofokeng [1,2], Suprakas Sinha Ray [1,2,*] and Vincent Ojijo [1]

[1] DST-CSIR National Centre for Nanostructured Materials, Council for Scientific and Industrial Research, Pretoria 0001, South Africa; TMofokeng@csir.co.za (T.G.M.); VOjijo@csir.co.za (V.O)

[2] Department of Applied Chemistry, University of Johannesburg, Doornfontein 2028, South Africa

* Correspondence: rsuprakas@csir.co.za or ssinharay@uj.ac.za; Tel.: +27-12-841-2388

Table S1. Amount of polymers, compatibilizers and clay present in the extruded samples.

Sample	Mass of PP (g)	Mass of LDPE (g)	Mass of PE-*g*-MA (g)	Mass of PP-*g*-MA (g)	Mass of Clay (g)	Mass of PP (wt %)	Mass of LDPE (wt %)	Mass of PE-*g*-MA (wt %)	Mass of PP-*g*-MA (wt %)	Mass of Clay (wt %)	Mass Fraction of PP	Mass Fraction of LDPE	TOTAL Mass Fraction of PP in the Sample	TOTAL Mass Fraction of LDPE in the Sample
100/0/0/0/0	2000	0	0	0	0	100	0	0	0	0	1	0	1	0
96/0/0/0/4	1920	0	0	0	80	96	0	0	0	4	0.96	0	0.96	0
0/100/0/0/0	0	2000	0	0	0	0	100	0	0	0	0	1	0	1
0/96/0/0/4	0	1920	0	0	80	0	96	0	0	4	0	0.96	0	0.96
80/20/0/0/0	1600	400	0	0	0	80	20	0	0	0	0.8	0.2	0.8	0.2
80/20/0/0/4	1536	384	0	0	80	76.8	19.2	0	0	4	0.768	0.192	0.768	0.192
80/20/5/0/4	1456	364	0	100	80	72.8	18.2	0	5	4	0.728	0.182	0.778	0.182
80/20/0/5/4	1456	364	100	0	80	72.8	18.2	5	0	4	0.728	0.182	0.728	0.232
80/20/5/5/4	1376	344	100	100	80	68.8	17.2	5	5	4	0.688	0.172	0.738	0.222

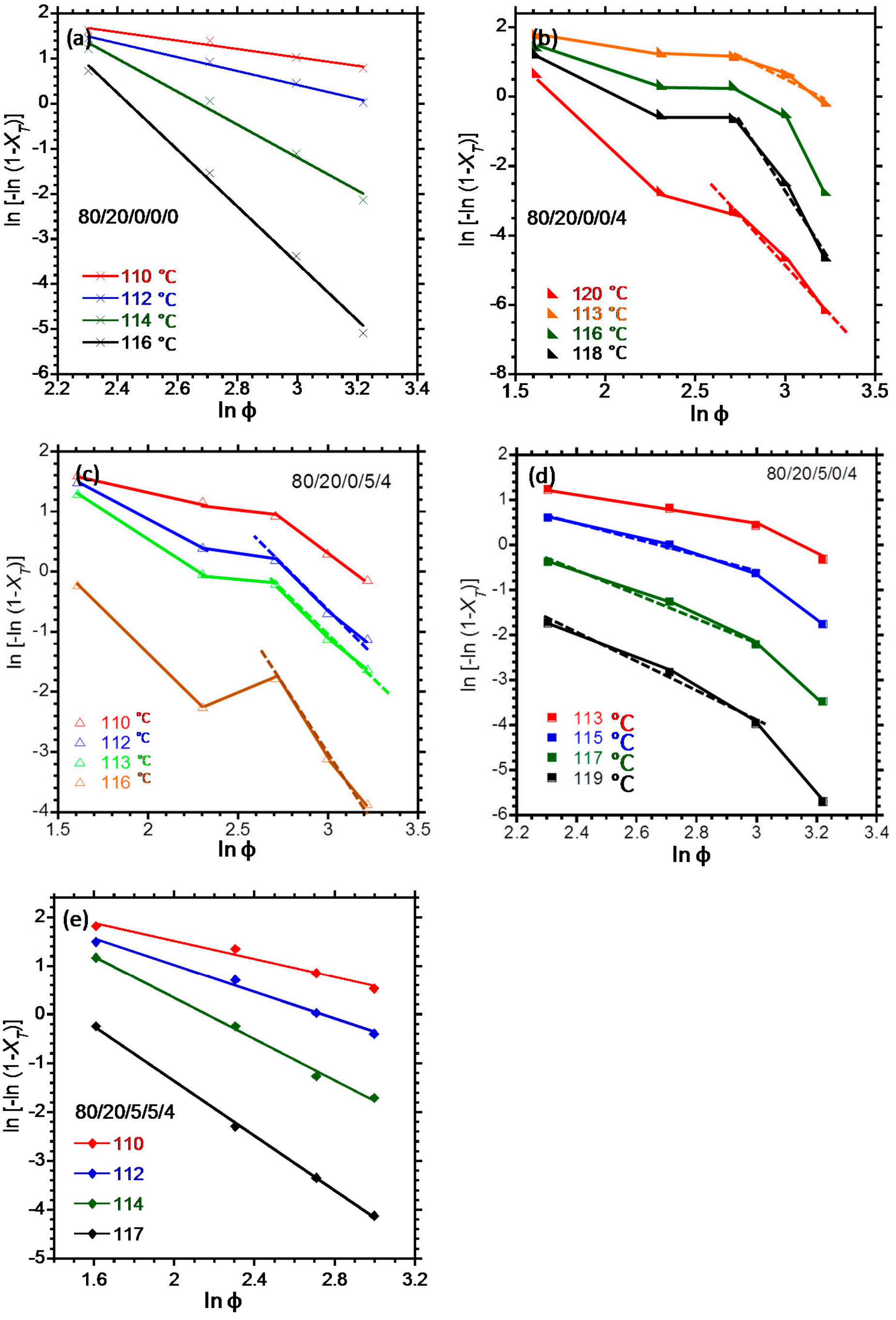

Figure S1. $\ln[-\ln(1-X_T)]$ versus $\ln\phi$ plots for: (**a**) neat blend and (**b**–**e**) blend composites.

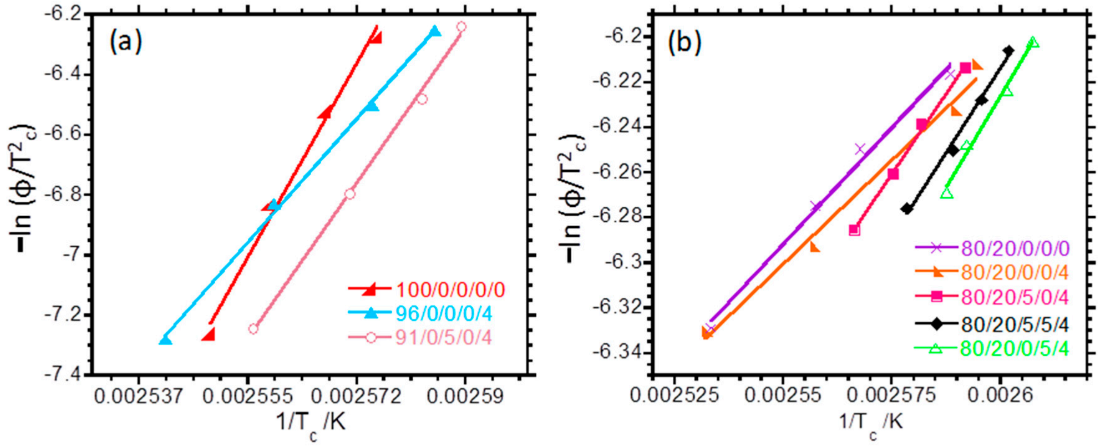

Figure S2. Determination of the activation energy, *ΔE* describing the nonisothermal crystallization process of PP in neat PP, blend, PP containing binary and ternary composites.

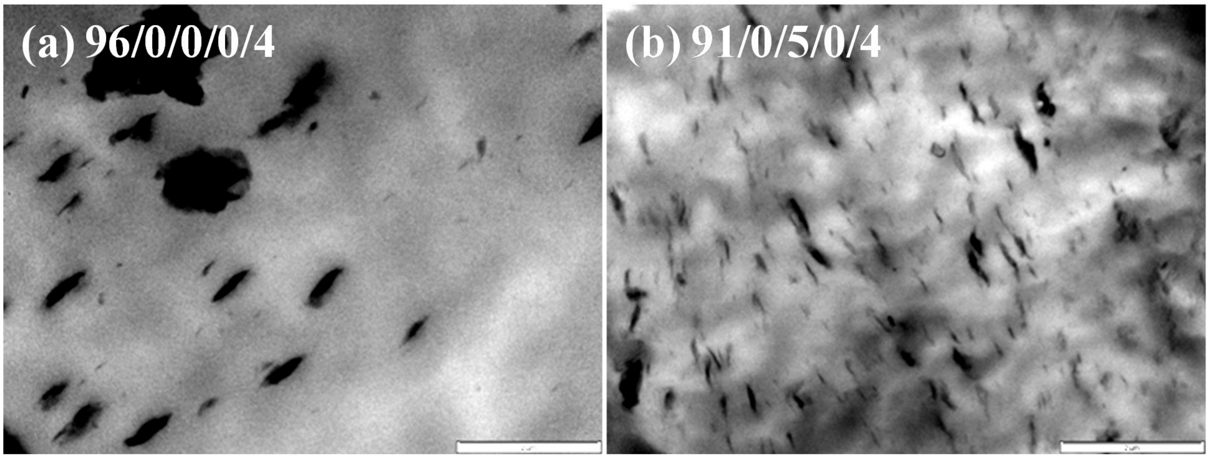

Figure S3. TEM micrographs of binary PP/LDPE/PP-*g*-MA/PE-*g*-MA/clay composites.

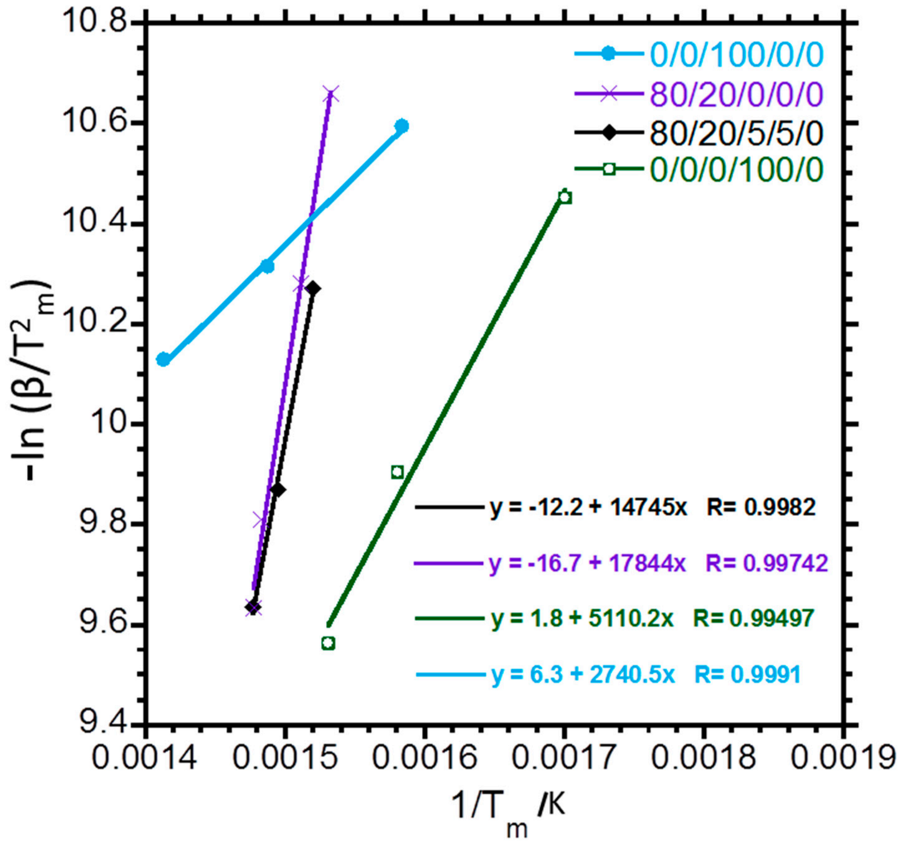

Figure S4. Determination of the activation energy, E_a describing the thermal degradation process of the neat blend, PP-*g*-MA, PE-*g*-MA, PP-*g*-MA and PE-*g*-MA containing blend.

Table S2. Activation energy for the overall non-isothermal crystallization of PP-*g*-MA, PE-*g*-MA, PP/LDPE, and PP/LDPE/ PP-*g*-MA/PE-*g*-MA blend.

**PP/LDPE/PP-*g*-MA/PE-*g*-MA/clay**	**Activation energy (E_a)/kJ·mol^{-1}**
80/20/0/0/0	148.4
0/0/100/0/0	22.8
0/0/0/100/0	42.5
80/20/5/5/0	122.6
